# Case report: rapid and durable response to PDGFR targeted therapy in a child with refractory multiple infantile myofibromatosis and a heterozygous germline mutation of the *PDGFRB* gene

**DOI:** 10.1186/s12885-017-3115-x

**Published:** 2017-02-10

**Authors:** Peter Mudry, Ondrej Slaby, Jakub Neradil, Jana Soukalova, Kristyna Melicharkova, Ondrej Rohleder, Marta Jezova, Anna Seehofnerova, Elleni Michu, Renata Veselska, Jaroslav Sterba

**Affiliations:** 1 Department of Pediatric Oncology, University Hospital Brno and School of Medicine, Masaryk University, Cernopolni 9, Brno, 613 00 Czech Republic; 2 Central European Institute of Technology, Masaryk University, Kamenice 753/5, Brno, 625 00 Czech Republic; 3 Laboratory of Tumor Biology, Department of Experimental Biology, School of Science, Masaryk University, Kotlarska 2, Brno, 611 37 Czech Republic; 4 Division of Medical Genetics, Department of Biology, University Hospital Brno and School of Medicine, Masaryk University, Cernopolni 9, Brno, 613 00 Czech Republic; 5 Department of Pathology, University Hospital Brno and School of Medicine, Masaryk University, Cernopolni 9, Brno, 613 00 Czech Republic; 6 Department of Pediatric Radiology, University Hospital Brno and School of Medicine, Masaryk University, Cernopolni 9, Brno, 613 00 Czech Republic; 7 International Clinical Research Center, St. Anne’s University Hospital Brno, Pekarska 53, Brno, 656 91 Czech Republic

**Keywords:** Infantile myofibromatosis, Tyrosine kinase inhibitor, PDGFR, Chemotherapy, Theranostics, Case report

## Abstract

**Background:**

Infantile myofibromatosis belongs to a family of soft tissue tumors. The majority of these tumors have benign behavior but resistant and malignant courses are known, namely in tumors with visceral involvement. The standard of care is surgical resection. Observations suggest that low dose chemotherapy is beneficial. The treatment of resistant or relapsed patients with multifocal disease remains challenging. Patients that harbor an actionable mutation in the kinase domain are potential subjects for targeted tyrosine kinase inhibitor therapy.

**Case presentation:**

An infant boy with inborn generalized infantile myofibromatosis that included bone, intracranial, soft tissue and visceral involvement was treated according to recent recommendations with low dose chemotherapy. The presence of a partial but temporary response led to a second line of treatment with six cycles of chemotherapy, which achieved a partial response again but was followed by severe toxicity. The generalized progression of the disease was observed later. Genetic analyses were performed and revealed a *PDGFRB* gene c.1681C>A missense heterozygous germline mutation, high PDGFRβ phosphokinase activity within the tumor and the heterozygous germline Slavic Nijmegen breakage syndrome 657del5 mutation in the *NBN* gene. Targeted treatment with sunitinib, the PDGFRβ inhibitor, plus low dose vinblastine led to an unexpected and durable response without toxicities or limitations to daily life activities. The presence of the Slavic *NBN* gene mutation limited standard chemotherapy dosing due to severe toxicities. Sister of the patient suffred from skull base tumor with same genotype and histology. The same targeted therapy led to similar quick and durable response.

**Conclusion:**

Progressive and resistant incurable infantile myofibromatosis can be successfully treated with the new approach described herein. Detailed insights into the biology of the patient’s tumor and genome are necessary to understand the mechanisms of activity of less toxic and effective drugs except for up to date population-based chemotherapy regimens.

**Electronic supplementary material:**

The online version of this article (doi:10.1186/s12885-017-3115-x) contains supplementary material, which is available to authorized users.

## Background

The family of fibroblastic-myofibroblastic tumors consists of more than 30 distinguished entities, such as inflammatory myofibroblastic tumor (IMT), aggressive fibromatosis and infantile myofibromatosis (IM). These tumors have uncertain biologic behaviors that range from low grade, locally aggressive and rarely metastasizing to a highly aggressive course that eventually evolves to a true high-grade sarcoma after recurrences. IM is a rare tumor that affects infants with a median age of 3 months; approximately 100 solitary lesion cases have been published in the literature during the past decade [1]. Soft tissue lesions of IM can arise at any time during life and, intriguingly, can regress spontaneously. However, visceral lesions are associated with high morbidity and mortality. The standard of care is the surgical resection of a single lesion. Multiple lesions and surgically unresectable lesions could be treated with anti-inflammatory drugs, interferon alpha, or distinct chemotherapeutic regimens that are based on low dose metronomic or maximum tolerated doses (MTD) of chemotherapeutics, such as the vinca alkaloids vincristine, vinorelbine and vinblastine; the alkylating agents cyclophosphamide and ifosfamide; or others, such as actinomycine D, doxorubicin or methotrexate [2–4]. The results of such treatments are under investigation in ongoing observational clinical trials of cooperative groups, such as European Soft Tissue Sarcoma Study Group (EpSSG) or Children’s Oncology Group (COG). Several studies of desmoid-type fibromatosis with response rates of 33–49% were reviewed elsewhere [4]. Nevertheless, the treatment of resistant patients, particularly those with visceral involvement, remains challenging.

For patients with progressive disease after MTD based chemotherapy, there are no established standards of care, and these patients are, thus, subjected to experimental treatments. One of the most promising agents with proven activity for IMT is the ALK tyrosine kinase inhibitor crizotinib [5]. Patients with ALK rearrangement are reportedly rapidly responding to crizotinib, but those without the detected fusion are not [5]. A recent work by Lovly et al. on IMTs revealed multiple fusion partners of ALK, and newly reported ROS1 and PDGFRβ fusions with projected TKI sensitivity were demonstrated in a patient with an ROS1 fusion [6]. Similar to IMTs, IMs may harbor missense mutations in the PDGFRβ kinase that constitutively alter PDGFR activity. Moreover, in several families, the c.1681C>T (p.Arg561Cys) mutation in the *PDGFRB* gene was found to cause familial infantile myofibromatosis [7]. A phase II study of sunitinib in 19 patients with aggressive fibromatosis has been published and described a 26.3% overall response, but the analysis of the kinase pathway was lacking [8]. A case report of aggressive fibromatosis that favored the PDGFRβ inhibitor sunitinib against imatinib was published that described a good response with sunitinib which was interrupted after 13 months and substituted by imatinib. But reactivation of painful lesions occurred within several days and re-growth of aggressive fibromatosis led to successful re-treatment with sunitinib [9].

Herein, we report the case of a patient with refractory multiple infantile myofibromatosis who was confirmed to harbor the *PDGFRB* germline mutation and who responded well to treatment with the PDGFRβ tyrosine kinase inhibitor sunitinib.

## Case presentation

The newborn boy with microtia and meatal atresia and with family history of two spontaneous missed abortions and myofibroblastic lesions with spontaneous regression in his older sister and father, was diagnosed with generalized myofibromatosis that affected the calva and radius bones, the spleen and subcutaneous tissue of face, the head, inguina and arm. Histopathology, with regard to the family history, revealed the presence of infantile familial myofibromatosis. Immunohistochemistry (ICH) and FISH did not reveal any pathological staining for ALK. The patient was treated according to the EpSSG 2005 observational trial recommendation with the metronomic vinblastine/methotrexate combination, which was expected to be less toxic than MTD based regimens. Despite this, severe neutropenia had been observed; therefore, a dose reduction was necessary down to 10%/30% of the original doses of vinblastine/methotrexate, respectively. The therapy was stopped after 8 weeks due to clearly progressive disease in the soft tissues and in the spleen and with the appearance of new FDG PET positive lesions in the bones. Thereafter, the standard MTD based therapy with vincristine/actinomycine D/cyclophosphamide – the “VAC” regimen with doses based on body weight (vincristine 0.05 mg/kg, actinomycine D 0.05 mg/kg, cyclophosphamide 50 mg/kg) had been initiated. Such treatment after the second course (the first course was given with a 75% reduction of cyclophosphamide) had led to severe febrile neutropenia, gastrointestinal toxicity with gastric palsy, subileus and bilateral bronchopneumonia. However, a reassessment after those 2 cycles revealed a partial response. Due to the previous toxicity, we decided to substitute vincristine with vinblastine at 10% of the recommended dose and cyclophosphamide at 75% of the recommended dose. The patient received the treatment without dose limiting toxicities up to six cycles and continued to respond. The patient was still in partial remission according to CT and MRI images and the FDG PET of the remaining measurable lesions was negative. Unfortunately, the first follow-up re-assessment confirmed the presence of progressive disease just 3 months after the last chemotherapy dose and several new lesions were detected in the humerus, head, lungs and skin, and all were FDG-PET positive.

**Fig. 1:**
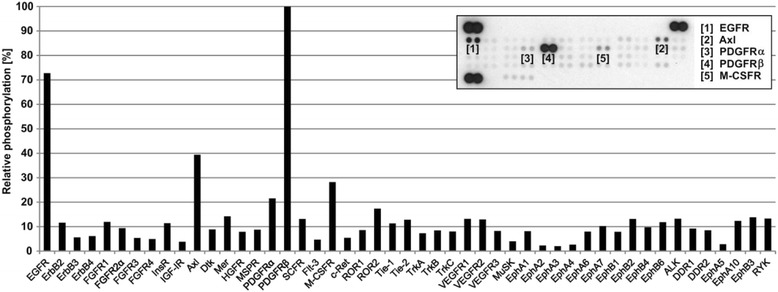
The relative phosphorylation of kinases in the tumor tissue sample

A new biopsy was carried out to obtain tumor tissue for phosphoproteomic analysis of the new lesion. The Human Phospho-RTK Array Kit was used to determine the relative levels of tyrosine phosphorylation of 49 different RTKs. The analysis was performed as previously described [10]. In addition to the antibodies (spotted in duplicate) against individual RTKs, each membrane contained three positive reference double spots and one negative control that was also spotted in duplicate and contained phosphate-buffered saline only. Furthermore, we also performed the following negative control experiment in each run: the membrane treated with lysis buffer only (without protein lysate) to ensure the specificity of the spotted antibodies. In such a design, a healthy control sample is not necessary for the determination of the RTK phosphorylation profile of the examined tumor tissue [11–13]. The phosphorylation profile of receptor tyrosine kinases showed that PDGFRβ kinase exhibited the highest level of activity and less intense positivity was observed for EGFR, M-SCFR, Axl and PDGFRα (Fig. 1). Targeted DNA analysis of the *PDGFRB* gene and next generation sequencing (NGS) were performed on genomic DNA from peripheral blood samples. We performed Sanger sequencing of the two *PDGFRB* regions to detect the presence of the c.1978C>A (p.Pro660Thr) and c.1681C>T (p.Arg561Cys) mutations [6] and uncovered a germ-line heterozygous c.1681C>A missense mutation that had previously been shown to be an IM causing mutation [14, 15]. To obtain the complex picture of the genetic background of the case we performed DNA analysis from peripheral blood with the Illumina TruSight Cancer panel, which enabled the sequencing of the hotspots in 94 predisposition cancer genes, according to the standard Illumina protocol (Illumina Inc., USA) and identified the heterozygous Slavic mutation 657del5 in the *NBN* gene of the NBS.

In the meantime, and based on parental request, the patient was observed for the next 4 months. He was doing very well clinically, with a Lansky performance status of 90% and with respect to his treatment history with toxicities after chemotherapy; we did not initiate another chemotherapy regimen but were awaiting the results of genetic analyses, which have revealed potential therapeutic targets. Further follow-up confirmed that the disease continued to progress; several new lesions were detected within the head and the left orbit, a new one was detected in the spine, and the spleen lesion had increased in size.

**Fig. 2:**
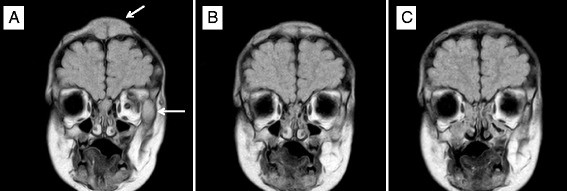
MRI Frontal view (seq. eFLAIR_long_TR_CLEAR). Two lesions of the left orbit and the skull in the fronto-parietal region (*bars*). **a** Before sunitinib treatment. **b** Day + 56 of sunitinib. **c** Day + 156 of sunitinib

**Fig. 3:**
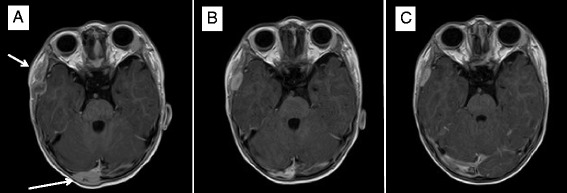
MRI Axial view (seq. esT1W_3S_FFE post-contrast). Intracranial lesions of the right temporal and right parieto-occipital regions (*bars*). **a** Before sunitinib treatment. **b** Day + 56 of sunitinib. **c** Day + 156 of sunitinib

**Fig. 4:**
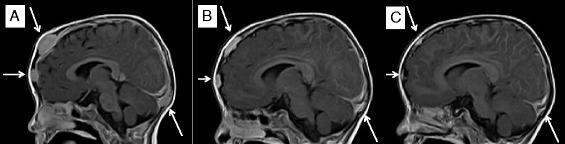
MRI Sagittal view (seq. esT1W_3S_FFE post-contrast). Frontal and parieto-occipital lesion (*bars*). **a** Before sunitinib treatment. **b** Day + 56 of sunitinib. **c** Day + 156 of sunitinib

Due to clear clinical and radiologic progression and new molecular genetic findings, and with respect to the history of the disease, we initiated the single agent *off-label* treatment with sunitinib 12.5 mg once a day. This dose corresponded to 2/3 of the recommended adult dose. An unexpected and dramatic reduction of the palpable soft tissue and bony lesions on the head was observed during the 4 weeks of treatment with the single agent sunitinib. An MR scan confirmed the regression of intracranial and intraorbital lesions as well (Figs. 2, 3 and 4). However, this dosing schedule led to grade 3–4 neutropenia, and the drug was stopped for 4 days. After only 4 days, we could observe the reactivation of the skin and soft tissue lesions; therefore, the sunitinib was given at the same dose every other day. Reactivated reddish swollen and painful sentinel lesions responded again to lower doses of sunitinib, but three more weeks of reduced doses of the single agent sunitinib did not lead to any further regression of the regressed but still palpable skin lesions. A low dose of vinblastine was added to the sunitinib. The starting vinblastine dose was 2 mg/m^2^; however, based on the further hematological toxicity, the dose was tapered down to a 0.4 mg/m^2^ dose once weekly.

An unexpected toxicity of sunitinib occurred after 4 months of treatment when accidental hypoglycemia led to a coma and the patient had to be admitted for glycemia corrections. Thereafter, the parents were educated on regular feeding before sunitinib administration. Further episodes of hypoglycemia were not noted. The patient remained on the treatment paradigm with a marked continuing response with no disease activity 1 year after the initiation of the treatment and without any dose limiting toxicities.

Interestingly, the 8 year old sister of the patient, who had a history of spontaneous regression of subcutaneous lesions, suffered from the symptomatic re-activation of the disease when the patient was receiving treatment. She presented with tumor size of 29 × 24 × 16 mm on the skull base with night pain. Histopathological and detailed mutation analyses found the same IM histopathology and the same genotype in the *PDGFB* and *NBN* genes. As with the index case, the sister is doing well on sunitinib and vinblastine treatment and has exhibited a rapid response. The nigh pain relieved after 2 weeks on sunitinib + vinblastine. Initial tumor volume shrinked by 44% after 97 days of combined treatment without any adverse events requiring reduction of doses. Timeline of both cases is shown on Additional file 1.

## Discussion and conclusions

Despite the finding that the patient exhibited a partial response to systemic VAC treatment, the disease continued to progress; moreover, the patient experienced severe, life threatening dose-limiting toxicities.

Inflammatory myofibroblastic tumors that harbor an ALK/ROS1 or PDGFRβ kinase fusion are potentially targetable with TKIs due to the presence of a constitutively active kinase domain that drives cellular proliferation [6, 16]. A response to the ALK inhibitor crizotinib is reported in tumors that harbor any of the ALK kinase fusions. Patients with IMT and ALK negative rearrangements are unlikely to respond to such targeted treatment.

*PDGFRB* mutations are reported to be involved in the pathogenesis of infantile myofibromatosis in a proposed autosomal dominant pattern with incomplete penetrance and variable expressivity [7]. The missense *PDGFRB* c.1681C>T (R681C) mutation is located in exon 12 and is predicted to decrease the autoinhibition of the JM domain (an autoinhibitory domain that masks the catalytic cleft when the receptor is not bound by its ligand) at baseline, which leads to increased kinase firing and promotes the formation of myofibromas in tissues with high PDGFRβ signaling activity. More recently, it was demonstrated in a cell culture model that the R561C mutation activates signaling pathways that are normally activated by the stimulated wild-type PDGFRβ receptor in the absence of PDGF [14]. PDGFR is the immediate NOTCH3 target gene [17]. If these two signaling pathways are linked and the IM disease-causing mutations in either *PDGFRB* or *NOTCH3* are demonstrated to be activating, theoretically, the inhibition of *PDGFRB* or *NOTCH3* would result in a targeted therapeutic strategy [7]. Our case report shows the clinical efficacy of such an approach. Targeted therapy against altered PDGFRβ with a TKIs inhibitor can overcome tumor growth and can lead to tumor shrinkage. Compared to the toxicity of conventional chemotherapy, treatment with sunitinib was tolerated well except for the occurrence of asymptomatic granulocytopenia and one episode of symptomatic hypoglycemia. However, the cessation of the drug lead to increased tumor activity and a decreased drug dose of the single agent sunitinib led to a stable disease only.

The analysis of tumor tissue or a patient’s samples and the use of a subsequent results driven treatment provide a new opportunity for personalized medicine as opposed to a population based study. Such treatments are supported by new insights into the molecular pathology of rare diseases, such as IM. A similar strategy would at least justify the *off-label* use of new drugs when the individual tumor biology and data about the safety of such drugs is well defined. TKIs could be an example, as these drugs are not available to orphan disease patients because of the absence of appropriate clinical trials. The careful management and regular observation of the patient is mandatory, however, in situations where standard approaches are either exploited or ineffective or absent, the prudent use of targeted agents based on the mechanism of action might lead to impressive results.

The rapid tumor re-growth that occurred when the patient was off of the sunitinib during the induction treatment indicates that metronomic dosing should be maintained at a lower dose with limited toxicity rather than being interrupted. The successful use of low dose vinblastine that is described here, together with the use of sunitinib at a dose of approximately 1/3 of the usually recommended dose per kg or m^2^ in adults, could be at least in part explained by the fact that targeted agents could act as biology response modifiers and lower doses of biological agents and chemotherapy could be nontoxic and advantageous [18, 19]. This theory is supported by our observation of the clear disease progression when sunitinib therapy was interrupted. Regular observations of the patient and preemptive measures such as the after-feeding dosing of sunitinib should be considered during treatment.

The finding of the Slavic mutation of the NBS was noted as accidental during NGS sequencing and the relevance for the disease course is unknown. The toxicity of chemotherapy might be at least in part conditioned by the NBS mutation As known, the intensity of chemotherapy in NBS patients must be adapted to individual risk factors and tolerance. The use of radiomimetics, alkylating agents, and epipodophyllotoxins should be avoided, and the dose of methotrexate should be limited [20].

However, the overall duration of such clinically effective treatment remains speculative, especially in patients with germline mutations. Different approaches that consider cancer to be a chronic disease, such as diabetes, should be considered in instances in which pathogenic germline mutations are in place. Should such targeted agents be maintained for a very long time, e.g., maintenance therapies in childhood acute leukemia, where other mechanisms of action, not only the cytostatic effect are in place? [21]. Should some pulses of targeted agents be considered?

These are only a few of the new questions that arose by the increased availability of diagnostic methods, such as NGS and functional proteomics.

The patients with an orphan disease like IM could benefit from detailed insights into the biology of their tumor and genome. Such approach is necessary to better understand the molecular pattern of disease and mechanisms of action of less toxic and effective drugs except for up to date population-based chemotherapy regimens. Morover, an unexpected finding of germline mutation can be important for treatment decisions. Progressive and resistant incurable infantile myofibromatosis can be successfully treated with the new approach described herein.

## Additional file

Additional file 1: Timeline. This file shows timeline of both described cases. (PDF 466 kb)

## Abbreviations

ALK: Anaplastic lymphoma kinase
COG: Children’s oncology group
EpSSG: European Soft Tissue Sarcoma Study Group
FDG PET: Fluorodeoxyglucose positron emission tomography
FISH: Fluorescent in situ hybridization
IHC: Immunohistochemistry
IM: Infantile myofibromatosis
IMT: Inflammatory myofibroblastic tumor
IVA: Ifosfamide/vincristine/actinomycine D
MRI: Magnetic resonance imaging
MTD: Maximum tolerated doses
MTX: Methotrexate
NBS: Nijmegen breakage syndrome
NGS: Next generation sequencing
PDGFR: Platelet derived growth factor receptor
PDGFRB: Platelet derived growth factor receptor gene B
PDGFRβ: Platelet derived growth factor receptor beta
TKI: Tyrosine kinase inhibitor
VAC: Vincristine/actinomycine D/cyclophosphamide
VBL: Vinblastine
