# Supplementary figures and images for: Case report: rapid and durable response to PDGFR targeted therapy in a child with refractory multiple infantile myofibromatosis and a heterozygous germline mutation of the PDGFRB gene
Source: BMC Cancer. 2017 Feb 10;17:119. doi: 10.1186/s12885-017-3115-x (PMC5301362; doi:10.1186/s12885-017-3115-x)

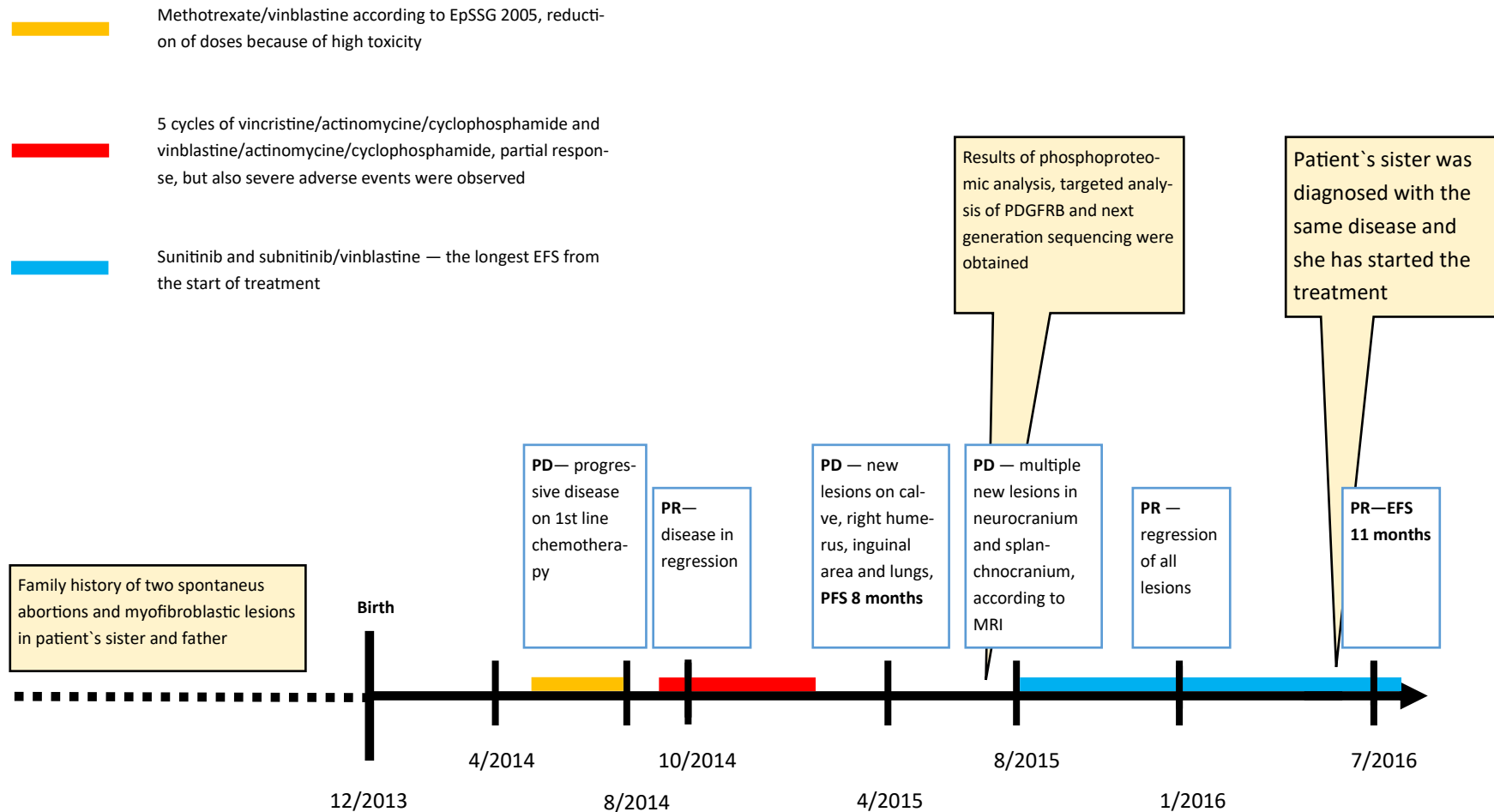

Supplement: Additional file 1: — Timeline. This file shows timeline of both described cases. (PDF 466 kb) [file 12885_2017_3115_MOESM1_ESM.pdf]
